# Localized Myxofibrosarcoma: A Retrospective Analysis of Primary Therapy and Prognostic Factors in 134 Patients in a Single Institution

**DOI:** 10.1093/oncolo/oyad332

**Published:** 2023-12-23

**Authors:** Athanasios Pogkas, Peter Reichardt, Per-Ulf Tunn, Maya Niethard, Mathias Werner, Saeed Ghani

**Affiliations:** Helios Klinikum Berlin Buch, Sarkomzentrum, Berlin, Germany; Vivantes Klinikum Neukölln, Berlin, Germany; Helios Klinikum Berlin Buch, Sarkomzentrum, Berlin, Germany; Medical School Berlin, Berlin, Germany; Helios Klinikum Berlin Buch, Sarkomzentrum, Berlin, Germany; Helios Klinikum Berlin Buch, Sarkomzentrum, Berlin, Germany; Helios Klinikum Berlin Buch, Sarkomzentrum, Berlin, Germany; MVZ Vivantes Friedrichshain Berlin, Berlin, Germany; Helios Klinikum Berlin Buch, Sarkomzentrum, Berlin, Germany

**Keywords:** myxofibrosarcoma, soft-tissue sarcoma, primary therapy, survival analysis, local recurrence, metastasis

## Abstract

**Background:**

Primary therapy of localized myxofibrosarcoma (MFS) remains controversial. Primary resection is complicated by a high rate of local recurrence, and the refractoriness to non-surgical treatment results in a higher risk of metastasis. The aim of the present study was to contribute the findings of a single sarcoma-specialized center and encourage investigating new treatment options.

**Patients and Methods:**

We analyzed 134 patients treated with localized MFS in our center regarding prognostic factors defining overall survival, local recurrence, and metastasis. We focused on multimodal treatment of localized MFS: surgery, radiation, chemotherapy, hyperthermia, and isolated limb perfusion.

**Results:**

The 5-year OS was 74.9%. From a total of 134 patients: 74 (55.2%) stayed disease free, 48 (35.8%) had a local recurrence (LR), and 23 (17.2%) developed a distant metastasis (DM). The 5-year LR-free survival (LRFS) and DM-free survival (DMFS) were 66.1% and 80.8%, respectively. Older age, tumor size (cT) cT ≥ 2, non-extremity localization, and distant metastasis were adverse predictive factors for OS. Performing an incision biopsy, surgery in a sarcoma-center, wide local excision or compartment-oriented excision, negative margins, and radiotherapy were positive predictive factors for LR. Tumor size cT ≥ 3 was a negative predictive factor for DM. Grading was a negative predictive factor for LR (G ≥ 2) and for DM (G3) in the multivariable analysis.

**Conclusion:**

Adjuvant radiation had a positive impact on LRFS in all localized tumor stages, even in cT1 tumors. Chemotherapy did not have a significant impact on DMFS, regardless of tumor stage. Our findings indicate that myxofibrosarcoma may be a chemotherapy-resistant entity and a much closer monitoring is required, in case of neoadjuvant treatment.

## Teaser

This retrospective analysis describes the experience of a single institution, focusing on the hypothesis that myxofibrosarcoma may be a chemotherapy-resistant entity.

**Implications for Practice**

Primary surgery treatment of localized myxofibrosarcoma should be performed in a sarcoma-specialized center. Current common practice does not embody adjuvant radiation of smaller tumors. The present analysis showed the positive impact of radiation on local recurrence, even in smaller tumors. No impact of chemotherapy was found, regarding metastasis, not even in larger tumors, regardless of combination with hyperthermia. Although common practice for larger tumors includes the use of chemotherapy, these data suggests that myxofibrosarcoma may be a chemotherapy-resistant entity. On that account, a much closer monitoring is necessary for these patients, to early detect a possible failure of neoadjuvant treatment.

## Introduction

Myxofibrosarcomas (MFS) are a subgroup of soft-tissue sarcomas (STS), which were classified separately in 2002 by the WHO^1^ and are described as a spectrum of malignant fibroblastic lesions with variably myxoid stroma and pleomorphism, possessing a distinctively curvilinear vascular pattern.^2,3^ According to epidemiological data in Germany, MFS represent ~5.8% of all sarcomas in men and 2.2% of all sarcomas in women with an age-standardized incidence rate of 0.2 and 0.1 per 100.000 per year, respectively.^4^ They occur mostly in the extremities of elderly patients.^5-8^

Primary resection is the mainstay of treatment, which is complicated by a high rate of local recurrence,^8-11^ and also by the refractoriness to non-surgical treatment, which results in a higher risk of metastasis.^12,13^

Single institutional observations have not been consistent, mostly due to their small sample^5,6,12,14-18^ and the lack of prospective data. Most studies provide evidence about general prognostic factors such as age, sex, tumor-size, grading, localization, and surgery characteristics such as type of surgery and resection margins. However, the impact of radiotherapy varies among studies^7,8,19,20^ and the sensitivity of MFS to chemotherapy remains controversial.^21^

The aim of this retrospective analysis was to contribute the experience of a single institution, focused on the hypothesis that MFS may be a chemotherapy-resistant entity. We therefore analyzed a cohort of patients treated in our clinic with regards to prognostic factors defining overall survival, recurrence, and metastasis. Our analysis was based on the outcome of primary multimodal treatment of localized MFS, including surgery, radiation, chemotherapy, regional hyperthermia, and isolated limb perfusion (ILP).

## Patients and Methods

### Patient Population

We identified a total of 142 patients which were diagnosed with MFS between 1998 and December 2020 in our Sarcoma Center, HELIOS Klinikum Berlin Buch. Prior to the data collection, we obtained an approval of the ethics committee of Berlin physicians’ council. Patients with primary distant or lymphatic metastasis, and with locally advanced disease (unresectable, not even with radical resection, or non-eligible for definitive radiation) were excluded (*n* = 8). Only patients with localized tumors were included (*n* = 134). All patients were adults at the time of diagnosis.

### Methods

All tumors were pathologically reviewed and classified as MFS through our pathology department or through a reference pathologist, according to the 2013 WHO classification of soft tissue and bone tumors.^2,3^ The assessment of tumor size before treatment (cT) was performed according to the Union for International Tumor Control and the American Joint Committee on Cancer (UIC/AJCC). The assessment of grading and resection margins were performed according to the French Federation of Cancer Centers Sarcoma Group (FNCLCC).

Where applicable, re-resections were performed after a R1 resection. If no R0 re-resection was performed, the margin status was classified as R1. Unplanned tumor resections with R2 or Rx resection margins that were followed by a planned surgery within a period of 3 weeks were classified as excision-biopsies. The minimum required dose of external photon beam radiation was 50 Gy for neoadjuvant, and 60 Gy for adjuvant intention. Most chemotherapy regimes were anthracycline based, in combination with another alkylating factor (ifosfamid or dacarbazine). One patient received monotherapy with trofosfamid, and one patient received monotherapy with gemcitabine. The minimum adequate dose for ifosfamid was 6 g/m^2^/cycle. Grade 1 tumors were not treated with chemotherapy.

Regional hyperthermia (with a temperature of 42 °C for a 60-minute period) was given concurrently with chemotherapy on 2 days of each cycle. The use of hyperthermia was according to the guidelines of the European Society for Hyperthermic Oncology.^22,23^

ILP was performed with a dose of 1-4 mg of TNF and 10-13 mg/L of limb-volume melphalan.^24-26^

Specialized sarcoma centers were defined as the ones listed by the German Society of Cancer (Deutsche Krebsgesellschaft).^27^

### Statistical Analysis

Overall survival (OS) was calculated from initial diagnosis until date of death (due to any cause). OS was censored at the date of last follow-up. Local recurrence-free survival (LRFS) was calculated from the date of treatment until the date of the first LR. Distant metastasis-free survival (DMFS) was calculated from the date of treatment until the date of DM. Both LRFS and DMFS were censored at the date of last follow-up or death.

Univariable survival analysis was conducted using the Kaplan-Meier method. The survival distributions were compared using the Log-rank test, both pooled and pairwise.

Multivariable analysis was conducted using Cox regression models. The results were presented as an adjusted hazard ratio (HR) with 95% confidence intervals (CI). For each model, we performed following statistics: –2LL, the likelihood-ratio and the overall chi-square. *P*–value of <0.05 was considered statistically significant.

We performed a separate explorative analysis of 2 subgroups. The first consisted of cT1 tumors regarding the effect of radiation on the LRFS, and the second consisted of cT ≥ 3 tumors regarding the effect of chemotherapy on the DMFS.

All statistics were performed with SPSS (IBM Corp. Released 2020. IBM SPSS Statistics for Macintosh, Version 27.0. Armonk, NY: IBM Corp).

## Results

### Patient Characteristics

The overview of the patient characteristics is demonstrated in Table 1. We analyzed a total of 134 patients, with mean age at diagnosis of 65 years (SD = 14). Follow-up range was 12-305 months with a median of 64.5 months. Five patients were lost to follow up. In some cases, it was not possible to retrospectively retrieve exact information, so we, respectively, excluded the missing data from the related analysis: in 19 cases the cT stage at diagnosis, in 39 cases the type of biopsy, in 9 cases the resection margins, in one case the grading, in 3 cases the site, where the surgery was performed, in 4 cases the type of surgery.

**Table 1.** Patient characteristics (LR: local recurrence, DM: distant metastasis, ILP: isolated limb perfusion).

Factor	No.	%
Outcome groups		
No LR, no DM	74	55.2
LR	37	27.6
DM	13	9.7
First LR + later DM	7	5.2
Synch LR + DM	3	2.2
Total	134	
Local recurrence		
No	86	64.2
Yes	48	35.8
Total	134	
Distant metastasis		
No	111	83.6
Yes	23	17.2
Total	134	
Sex		
Male	73	54.5
Female	61	45.5
Total	134	
Age groups, years		
≤59	47	35.1
60-79	67	50.0
≥80	20	14.9
Total	134	
Tumor size (cT)		
1	34	29.6
2	47	40.9
3	22	19.1
4	12	10.4
Total	115	
Grade		
1	12	9.0
2	69	51.9
3	52	39.1
Total	133	
Localization		
Lower extremity	74	55.2
Upper extremity	32	23.9
Pelvic region	11	8.2
Thorax	12	9.0
Other	5	3.7
Total	134	
Localization		
Extremity	106	79.1
Other	28	20.9
Total	134	
Biopsy prior to surgery		
No	39	29.1
Yes	95	70.9
Total	134	
Clinic of biopsy		
Non-specialists	57	60.0
Sarcoma Center	38	40.0
Total	95	
Type of biopsy		
Incision	54	56.8
Excision	35	36.8
Core	6	6.3
Total	95	
Surgery		
No	2	1.5
Yes	132	98.5
Total	134	
Clinic of surgery		
Non-specialists	35	26.7
Sarcoma center	96	73.3
Total	131	
Resection-status (R)		
R0	90	72.0
R1	33	26.4
R2	2	1.6
Total	125	
Type of surgery		
Local non-wide excision	36	27.7
Wide local excision	60	46.2
Compartment-oriented excision	28	21.5
Amputation	6	4.6
Total	130	
ILP		
No	126	94.0
Yes	8	6.0
Total	134	
Radiation		
No	49	36.6
Yes	85	63.4
Total	134	
Intention of radiation		
Neoadjuvant	7	8.2
Adjuvant	76	89.4
Definite	2	2.4
Total	85	
Radiation + hyperthermia		
No	79	92.9
Yes	6	7.1
Total	85	
Chemotherapy		
No	104	77.6
Yes	30	22.4
Total	134	
Intention of chemotherapy		
Neoadjuvant	20	66.7
Adjuvant	10	33.3
Total	30	
Chemotherapy + hyperthermia		
No	15	50.0
Yes	15	50.0
Total	30	

Two patients did not receive surgery and were treated with definitive radiation. The majority of patients who received chemotherapy had at least 3 or more cycles (*n* = 27, 93.1%).

From a total of 134 patients: 74 (55.2%) stayed disease free; 37 patients (27.6%) had a local recurrence (LR), while 7 patients (5.2%) had first an LR and developed later metastasis; 13 patients (9.7%) developed metastasis without an LR; 3 patients (2.2%) developed synchronously a LR and metastasis.

### Overall Survival

**Figure 1.**
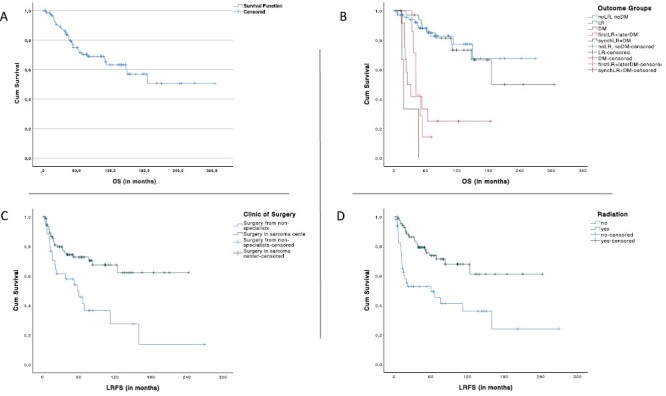
Univariate analysis regarding overall survival (OS) and local recurrence-free survival (LRFS). (**A**) Overall survival in months. (**B**) Kaplan-Meier curve regarding outcome groups and OS, *P* < .001. (**C**) Kaplan-Meier curve regarding clinic of surgery and LRFS, *P*= .001. (**D**) Kaplan-Meier curve regarding radiation and LRFS, *P* < .001 (LR: local recurrence, DM: distant metastasis).

Thirty-nine patients died (30.2%) from a total of 129 (5 lost in follow up). The 2- and 5-year OS survival (5yOS) were 90.8% and 74.9%, respectively (Fig. 1A). The univariate analysis results are demonstrated in Supplementary Table S1.

The 5yOS for patients ≥80 years old was 49.4%, for patients between age of 60-79 years old was 76.4% and for patients of age ≤59 years old was 82.8% (*P*= .001). Patients with distant metastasis had a worse 5yOS (87.7% vs 22.7%, *P* < .001; Fig. 1B). The 5yOS for cT ≥ 3 tumors (44.2%) was worse than tumors with cT ≤ 2 (85.4%; *P*= .001). The pairwise analysis showed no significance between cT1 (84.2%) and cT2 (86.2%) tumors (*P*= .711), or between cT3 (55.4%) and cT4 (25%) tumors (*P*= .187).

The 5yOS of extremity tumors was better than the other localizations (79.7% vs 56.5%, *P*= .056). The 5yOS of lower extremity tumors (80.4%) was better than other localizations (*P*= .028); however, in the pairwise analysis, the difference was significant only between lower extremity and pelvic region tumors (80.4% vs 42.0%; *P*= .003). Amputated patients (*n* = 6) had the worst 5yOS outcome (50%); in the pairwise analysis the 5yOS for amputation (50%, *n* = 6) was worse than the wide local excision (76.7%, *n* = 60, *P*= .030) and worse than the compartment-oriented excision (76.7%, *n* = 25, *P*= .039). Adjuvant radiation was associated with a better 5yOS (75.7%, *n* = 74) than neoadjuvant radiation (53.3%, *n* = 7) and better than definitive radiation (0.0%, *n* = 2; *P* < .001).

The multivariable analysis results are demonstrated in Table 2. Significant risk factors were age from 60 to 79 years (HR = 2.96, *P*= .041) and age ≥ 80 years (HR = 6.02, *P*= .003; compared to age ≤ 59 years), distant metastasis (HR = 5.43, *P* < .001), and non-extremity localization (HR = 2.54, *P*= .042).

**Table 2.** Multivariable Cox regression models for OS, LRFS, and DMFS.

Factor (OS)	HR OS	95% CI (OS)	*P*-value	Factor (LRFS)	HR LRFS	95% CI (LRFS)	*P*-value	Factor (DMFS)	HR DMFS	95% CI (DMFS)	*P*-value
Age ≤ 59	Ref			cT ≤ 2	Ref			cT ≤ 2	Ref		
Age 60 – 79	2.96	1.04-8.42	**.041**	cT ≥ 3	1.82	0.65-5.02	.248	cT ≥ 3	7.51	2.65-21.24	**<.001**
Age ≥ 80	6.02	1.82-19.87	**.003**								
				Grade = 1	Ref			Grade ≤ 2	Ref		
DM no	Ref			Grade ≥ 2	6.26	1.34-29.26	**.020**	Grade = 3	3.37	1.29-8.82	**.013**
DM yes	5.43	2.46-11.99	**<.001**								
				Biopsy yes	Ref			Surgery in SC	Ref		
cT ≤ 2	Ref			Biopsy no	0.26	0.06-1.08	.064	Surgery not in SC	0.95	0.19-4.72	.950
cT ≥ 3	1.94	0.82-4.55	.128								
				Surgery in SC	Ref			Radiation yes	Ref		
Grade ≤ 2	Ref			Surgery not in SC	3.13	0.81-12.03	.096	Radiation no	0.30	0.63-4.21	.308
Grade = 3	1.54	0.73-3.22	.248								
				R0	Ref			Chemo yes	Ref		
Loc extremity	Ref			*R* ≥ 1	2.43	1.03-5.70	**.041**	Chemo no	2.31	0.70-7.62	.169
Loc other	2.54	1.03-6.26	**.042**								
				WLE	ref						
Radiation yes	Ref			LNWE	3.76	1.13-12.50	**.031**				
Radiation no	1.06	0.48-2.34	.884	COE	0.50	0.14-1.76	.285				
				Amputation	0.31	0.03-2.76	.298				
Chemo yes	Ref										
Chemo no	0.51	0.19-1.35	.178	Radiation yes	Ref						
				Radiation no	7.85	3.14--19.62	**<.001**				
				Chemo yes	Ref						
				Chemo no	0.85	0.32--2.26	.750				

HR: hazard ratio, CI: confidence interval, OS: overall survival, LRFS: local recurrence-free survival, DMFS: distant metastasis free survival, DM: distant metastasis, cT: tumor size before treatment, Loc: localization, R: resection margins, SC: sarcoma center, WLE: wide local excision, LNWE: local non-wide excision, COE: compartment-oriented excision.

### Local Recurrence-Free Survival

Forty-eight patients (35.8%) from a total of 134 had an LR. The 2- and 5-year LRFS (5yLRFS) was 75.3% and 66.1%, respectively. Univariate analysis results are demonstrated in Supplementary Table S2.

Only 3 of 12 G1 tumors had an LR. Performing a biopsy resulted in a better 5yLRFS (72.7% vs 50.9%, *P*= .032). Incision biopsy had a better 5yLRFS than the excision biopsy (76.1% vs 63.6%, *P*= .071), which was pairwise significant (*P* = .037). Patients operated in a sarcoma center had a better 5yLRFS than patients operated from non-specialists (72.7% vs 49.9%, *P*= .001; Fig. 1C). Local non-wide excision had a worse 5yLRFS than wide local excision (42.0% vs 71.9%, *P* < .001) and worse than compartment-oriented excision (42.0% vs 83.9%, *P* < .001). Surgery with R0 margins had a better 5yLRFS than with *R* ≥ 1 (74.8% vs 48.0%, *P*= .002). Radiotherapy had a positive impact on the 5yLRFS (73.6% vs 52.7%, *P* < .001; Fig. 1D). Neoadjuvant radiation (*n* = 7) showed a worse 5yLRFS than the adjuvant radiation (*n* = 76; 60% vs 75.3%, *P*= .049).

The multivariable analysis results are demonstrated in Table 2. Significant risk factors were Grade ≥ 2 (HR = 6.26, *P*= .020), resection margins *R* ≥ 1 (HR = 2.43, *P*= .041), local non-wide excision (compared to wide local excision; HR = 3.76, *P*= .031), and no-radiation (HR = 7.85, *P* < .001).

### Distant Metastasis-Free Survival

From a total of 134 patients, 23 (17.2%) developed distant metastases. The 2- and 5-year DMFS (5yDMFS) was 89.7% and 80.8%, respectively. The most prevalent sites of primary metastasis were the lungs (*n* = 11, 47.8%) and lymph nodes (*n* = 4, 17.4%). Remaining sites of metastasis are demonstrated in Supplementary Table S3. Univariate analysis results are demonstrated in Supplementary Table S2.

Grade 1 tumors developed no metastases. Smaller tumors (cT ≤ 2) had a better 5yDMFS than larger ones (cT ≥ 3) (89.1% vs 54.2%, *P* < .001). Patients operated in a sarcoma center had a worse 5yDMFS than the ones operated by non-specialists (94.2% vs 75.9%, *P*= .054).

The multivariable analysis results are demonstrated in Table 2. Significant risk factors were cT ≥ 3 (HR = 7.513, *P* < .001) and grade 3 (HR = 3.375, *P*= .013).

### Subgroups

**Figure 2.**
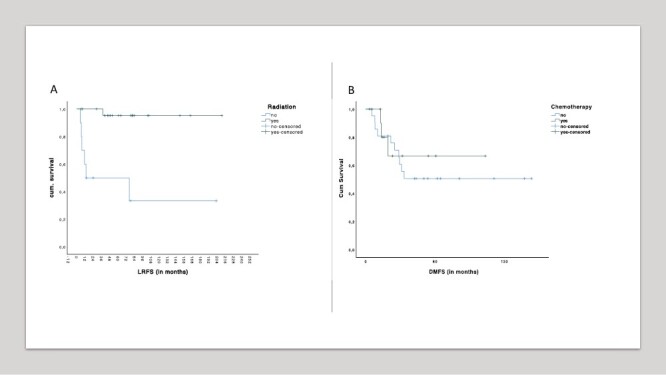
Subgroups univariate analysis. (**A**) Kaplan-Meier curve regarding radiation on cT1 tumors and LRFS, *P* < .001. **(B)** Kaplan-Meier curve regarding chemotherapy on cT ≥ 3 tumors and DMFS, *P*= .922 (LRFS: local recurrence-free survival, DMFS: distant metastasis free survival).

Irradiated cT1 tumors had a better 5yLRFS (95% vs 33%, *P* < .001; Fig. 2A, Table 3). Thereof, only 7/34 patients (20.6%) had an *R* ≥ 1 resection and only 3/34 (8.8%) had a G1 tumor. The Cox regression univariate analysis showed a significantly higher risk (HR = 21.223, *P*= .005) for the non-irradiated ones.

**Table 3.** Kaplan-Meier and univariate Cox regression analysis: radiotherapy in cT1 tumors regarding local recurrence-free survival (LRFS), and chemotherapy in cT ≥ 3 tumors regarding distant metastasis-free survival (DMFS) (HR: hazard ratio, CI: confidence interval, cT: tumor size before treatment).

Factor	Total	Events	2 years %	5 years %	*P*-value	HR	95% CI	*P*-value
Radiotherapy in cT1 tumors			LRFS	LRFS	<.001			
Yes	24	1	100.0	95.2		Ref		
No	10	6	50.0	33.3		21.22	2.50--179.58	**.005**
Chemotherapy in cT ≥ 3 tumors			DMFS	DMFS	.617			
Yes	13	3	66.7	66.7		Ref		
No	21	10	75.9	50.6		1.39	0.37--5.12	.618

The analysis of the cT ≥ 3 subgroup (*n* = 34; Fig. 2B, Table 3) showed no impact of chemotherapy on the DMFS (HR = 1.392, *P*= .618; Fig. 2B, *P*= .617). Thereof, only 2/34 (5.9%) had a G1 tumor and 12/34 (35.3%) had a G3 tumor. The univariable Cox analysis also showed no statistical significance regarding the risk of the cT ≥ 3 tumors that were not treated with chemotherapy (HR = 1.392, *P*= .618).

## Discussion

Myxofibrosarcoma is classified as a distinct histologic entity since 2002 after a series of studies indicating the necessity for a differentiation from the so-called malignant fibrous histiocytoma (MFH).^11^ Until then, the term Myxofibrosarcoma was inconsistently used in literature to characterize malignant soft-tissue tumors, which had not been defined as a specific entity.

In our study, the OS at 2 and 5 years was 90.8% and 74.9%, respectively. Previous studies with a population of at least 100 patients, which were published from 2011 to 2022 report a 5-year OS, which varies from 68% to 84%.^7,8,13,28,29^ Our study highlights adverse prognostic factors for OS such as older age, large tumor size (cT ≥ 3), localization (pelvis and non-extremity), and occurrence of metastases, which is in accordance with previous reports.^8,13,28-31^ However, in contrast to these reports, we did not detect significant differences regarding the impact of grading, margin status, clinic of surgery and adjuvant radiotherapy on OS. These factors may play an important role indirectly by affecting metastasis or LR. We found that high grade tumors (G3) had a risk to develop metastasis and that low-grade tumors (G1) developed no metastases. LR did not affect OS; however, achieving local control is of great importance, since MFS can recur in higher grading, imposing a greater risk for metastasis.^11^

In our study, the LR rate at 2 and 5 years was 24.7% and 33.9% accordingly. In previous reports, the 5-year LR rate varies from 19.1% to 32.7%.^7,32^ We found grade ≥ 2, no-biopsy, excision biopsy, surgery in a non-sarcoma-center, non-wide local excision, positive margins (*R* ≥ 1), and no-radiation to be negative prognostic factors regarding LR. Grading was a risk factor in the multivariate analysis (G ≥ 2); however, the univariate analysis showed no statistical difference [only 3/12 (25%) of G1 tumors had an LR]. Patients surgically managed at sarcoma centers seem to have a better 5yLRFS; however, this finding was significant only in the univariate analysis, not in the multivariant Cox regression model.

Larger tumors tend to recur more frequently, since the complexity and extent of surgery can lead to inadequate resection. Out of 30 patients with cT ≥ 3, 11 patients (36.7%) had an *R* ≥ 1 resection. Local non-wide excision had both in the univariate and multivariable analysis a high risk for recurrence. There is extensive evidence regarding the infiltrative, untypical growth pattern of MFS along the fasciae with a lack of pseudocapsule (a so-called MRI tail-sign) that makes it difficult to determine the extent of the tumor during surgery.^33-35^ Our data suggest that wide local excision or compartment-oriented excision should primarily be considered as surgery options for MFS. Amputated patients (*n* = 6) had the worst survival outcome. Two of those patients developed a metastasis, 2 patients had a cT ≥ 3 tumor and 4 were 60-79 years old. Due to the small number of amputated patients, we cannot deliver a valid result regarding survival.

Incision biopsies or core-needle biopsies are acceptable diagnostic options for STS.^36,37^ Our data confirm these findings, since biopsy itself, and in particular incision-biopsy was a positive predictive factor for LRFS. Kikuta et al described the impact of previous unplanned excisions to be the most important negative predictor regarding LR, in a series of 64 patients.^38^ Although not significant enough, our data suggest, that unplanned excisions are negatively related to LRFS.

Radiotherapy was performed in the adjuvant setting for the majority of cases (89.4%) and was combined with hyperthermia in 6 cases (7.1%). Our findings showed the importance of radiotherapy achieving a better LRFS. Previous studies also highlighted this matter.^7,13,15,19^ Patients with smaller tumors (cT1) also benefited from radiotherapy. This finding is of considerable importance, since radiotherapy is not part of the common practice in the treatment of cT1 STS.

Our findings regarding DMFS correspond to the nomogram of Callegaro et al in which myxofibrosarcoma was the histotype among STS with the best prognosis in terms of distant metastases.^39^ Italiano et al described in a multivariate analysis of the French Sarcoma Group Database that only no-MFH histology had a worse DMFS and OS.^40^ We found tumor-size >10 cm (cT ≥ 3) and grade 3 to be negative prognostic factors for DM. Regarding the clinic of surgery, our data showed a worse DMFS for tumors operated in a sarcoma-center, but these cases were unfavorably selected: they were larger (*n* = 30, 96.8% of the cT ≥ 3 tumors), had a higher grade (*n* = 39, 76.5% of the G3 tumors) and they had a higher rate of previous unplanned biopsies (*n* = 28, 82.4% of unplanned biopsies). Recurrence was not an independent factor regarding metastasis. However, we believe that local relapse in our study did not influence the DMFS, possibly because there were not enough G3 MFS upfront (39%). As already mentioned, local control is essential, since MFS has the tendency to recur in higher grades, subsequently posing a greater risk for metastasis.^11^

The use of chemotherapy as part of the primary therapy in localized, non-advanced MFS has its evidence in a long series of studies examining all types of STS. The European Sarcoma Network Working Group (ESMO) suggests its use in high-risk patients (high grade, deep, >5 cm tumors).^41^ In the most prospective studies regarding adjuvant chemotherapy in high grade STS, MFS were not included as a distinct histologic entity. In the results of the Italian Randomized Cooperative Trial in 2001^42^ and in the study of Frustaci et al on behalf of the Italian Sarcoma Group in 2003,^43^ malignant fibrous histiocytoma was included among other high-grade sarcomas as an undifferentiated pleomorphic entity. Nevertheless, the studies did provide evidence of benefit on high-grade STS, which was later systematically reviewed in a meta-analysis of Pervaiz et al.^44^ In the EORTC 62931 trial of Woll et al,^45^ MFH was also not differentiated.

Regarding neoadjuvant chemotherapy, in the randomized phase II study of Gortzak et al, in which MFH represented 29% (*n* = 150) of cases without MFS differentiation, it was found that neoadjuvant chemotherapy with doxorubicin and ifosfamide did not compromise subsequent treatment (surgery with or without radiotherapy); however, it did not have enough power to draw definitive conclusions on benefit.^46^ In the randomized clinical trial of Gronchi et al,^47^ which provided the main evidence for the establishment of neoadjuvant chemotherapy on STS, the MFS were included among “other” histology types (*n* = 88, 26.83%). Also, in the long-term cohort follow-up, MFS were in the reference group of the multivariable analysis among undifferentiated-pleomorphic, and NOS Sarcomas.^48^ In the ISG-STS 1001 clinical trial, which compared the histotype-tailored neoadjuvant chemotherapy vs standard chemotherapy in patients with high-risk soft-tissue sarcomas, MFS were not included as a separate histologic entity.^49^

The majority of retrospective studies on MFS showed no benefit from chemotherapy or had a small sample.^8,19,20,28,29^ In the prognostic MFS nomogram of Cao et al,^30^ chemotherapy had a positive impact on OS; however, 21% of the patients had primary metastasis, relapsed tumors were not differentiated into local vs distant, and no data are presented regarding regimes and dosing. Look et al found a positive impact of chemotherapy regarding DMFS, but the sample of the study was very small: only 13/69 (18.8%) patients received chemotherapy (including 3 patients with G1 tumor) and only 7 patients received an anthracycline regime (MAID Schema).^17^ Colia et al analyzed retrospectively 34 patients with advanced MFS, which received chemotherapy with palliative intention. Their analysis suggests that the combination of anthracyclines and ifosfamide is active in myxofibrosarcoma and that in previously treated patients monotherapy with high-dose ifosfamide showed activity as well, thus the median PFS war only 4 months.^21^ In our study, we found no benefit of chemotherapy regarding OS, LRFS, or DMFS. Subgroup analysis of cT ≥ 3 tumors also revealed no benefit.

Hyperthermia (combined with chemotherapy or radiotherapy) had no impact on OS, LRFS, or DMFS. The evidence of combining chemotherapy with hyperthermia is based on the EORTC 62961/ESHO RHT-95 study,^22^ in which the MFS were just a small subcategory among “other sarcomas.” Our findings suggest the lack of benefit regarding combination of chemotherapy with hyperthermia; however, there is no clear evidence on this matter.

## Conclusion

Based on the above discussion, we may suggest that wide local excisions or compartment-oriented excisions should be the primary surgical options regarding MFS and should be performed in a sarcoma-specialized center. Adjuvant radiation should be considered part of the primary treatment, even for smaller tumors. Chemotherapy, if used as part of the primary therapy, should be very closely monitored, to exclude neoadjuvant treatment failure. We believe that MFS may be a chemotherapy-resistant entity. This matter should be a subject of more prospective, multicentered studies, incorporating the combination with regional hyperthermia.

## Supplementary Material

Supplementary material is available at *The Oncologist* online.

oyad332_suppl_Supplementary_Tables_1

oyad332_suppl_Supplementary_Tables_2

oyad332_suppl_Supplementary_Tables_3

## Data Availability

The data underlying this article will be shared on reasonable request to the corresponding author.
