# Supplementary material for: Localized Myxofibrosarcoma: A Retrospective Analysis of Primary Therapy and Prognostic Factors in 134 Patients in a Single Institution
Source: Oncologist. 2023 Dec 23;29(4):e544–52. doi: 10.1093/oncolo/oyad332 (PMC10994258; doi:10.1093/oncolo/oyad332)
Supplement: oyad332_suppl_Supplementary_Tables_1 [file oyad332_suppl_supplementary_tables_1.docx]

| **Factor** | **Total** | **Events** | **2-years OS %** | **5-years OS %** | **p-value** |
| --- | --- | --- | --- | --- | --- |
| **Outcome groups** |  |  |  |  | **<0,001** |
| no LR, no DM | 71 | 11 | 95,4 | 88,2 |  |
| LR | 36 | 10 | 97,1 | 88,0 |  |
| DM | 12 | 9 | 58,3 | 33,3 |  |
| first LR + later DM | 7 | 6 | 100,0 | 14,3 |  |
| synch LR+DM | 3 | 3 | 33,3 | 0,0 |  |
|  |  |  |  |  |  |
| **Local recurrence** |  |  |  |  | 0,240 |
| no | 82 | 19 | 90,6 | 79,8 |  |
| yes | 47 | 20 | 91,1 | 68,4 |  |
|  |  |  |  |  |  |
| **Distant metastasis** |  |  |  |  | **<0,001** |
| no | 107 | 21 | 96,0 | 87,7 |  |
| yes | 22 | 18 | 68,2 | 22,7 |  |
|  |  |  |  |  |  |
| **Sex** |  |  |  |  | 0,515 |
| Male | 69 | 22 | 89,1 | 75,9 |  |
| Female | 60 | 17 | 92,9 | 73,7 |  |
|  |  |  |  |  |  |
| **Age groups** |  |  |  |  | **0,001** |
| ≤59 | 47 | 7 | 90,9 | 82,8 |  |
| 60 – 79 | 64 | 22 | 93,1 | 76,4 |  |
| ≥80 | 18 | 10 | 83,3 | 49,4 |  |
|  |  |  |  |  |  |
| **Tumor size (cT)** |  |  |  |  | **0,001** |
| 1 | 34 | 9 | 94,0 | 84,2 |  |
| 2 | 45 | 10 | 92,7 | 86,2 |  |
| 3 | 20 | 7 | 82,4 | 55,4 |  |
| 4 | 12 | 7 | 70,0 | 25,0 |  |
|  |  |  |  |  |  |
| **Tumor size (cT)** |  |  |  |  | **0,001** |
| ≤2 | 79 | 19 | 93,3 | 85,4 |  |
| ≥3 | 32 | 14 | 77,9 | 44,2 |  |
|  |  |  |  |  |  |
| **Grade** |  |  |  |  | 0,214 |
| 1 | 12 | 2 | 100,0 | 90,9 |  |
| 2 | 65 | 17 | 91,6 | 80,7 |  |
| 3 | 52 | 20 | 87,5 | 64,2 |  |
|  |  |  |  |  |  |
| **Grade** |  |  |  |  | 0,364 |
| 1 | 12 | 2 | 100,0 | 90,9 |  |
| ≥2 | 117 | 37 | 89,8 | 69,9 |  |
|  |  |  |  |  |  |
| **Grade** |  |  |  |  | 0,092 |
| ≤2 | 77 | 19 | 93,0 | 82,0 |  |
| 3 | 52 | 20 | 87,5 | 64,2 |  |
|  |  |  |  |  |  |
| **Localization** |  |  |  |  | **0,028** |
| Lower Extremity | 71 | 15 | 93,8 | 80,4 |  |
| Upper Extremity | 31 | 13 | 93,5 | 78,6 |  |
| Pelvic Region | 11 | 6 | 70,0 | 42,0 |  |
| Thorax | 5 | 1 | 90,0 | 56,3 |  |
| Other | 11 | 4 | 80,0 | 80,0 |  |
|  |  |  |  |  |  |
| **Localization** |  |  |  |  | 0,056 |
| Extremity | 102 | 28 | 93,7 | 79,7 |  |
| Other | 27 | 11 | 79,5 | 56,5 |  |
|  |  |  |  |  |  |
| **Biopsy prior to surgery** |  |  |  |  | 0,565 |
| no | 36 | 12 | 88,6 | 79,7 |  |
| yes | 93 | 27 | 91,7 | 72,4 |  |
|  |  |  |  |  |  |
| **Clinic of biopsy** |  |  |  |  | 0,262 |
| Non-Specialists | 56 | 17 | 94,4 | 76,6 |  |
| Sarcoma Center | 37 | 10 | 87,0 | 63,9 |  |
|  |  |  |  |  |  |
| **Type of biopsy** |  |  |  |  | 0,335 |
| Incision | 53 | 17 | 87,2 | 69,4 |  |
| Excision | 34 | 9 | 96,9 | 73,0 |  |
| Core | 6 | 1 | 100,0 | 100,0 |  |
|  |  |  |  |  |  |
| **Clinic of surgery** |  |  |  |  | 0,396 |
| Non-Specialists | 34 | 10 | 91,2 | 84,9 |  |
| Sarcoma center | 92 | 27 | 92,7 | 71,8 |  |
|  |  |  |  |  |  |
| **Resection-status** |  |  |  |  | 0,681 |
| R0 | 88 | 25 | 92,6 | 77,2 |  |
| R≥1 | 33 | 11 | 90,6 | 69,8 |  |
|  |  |  |  |  |  |
| **Type of surgery** |  |  |  |  | 0,138 |
| Local non-wide excision | 34 | 11 | 90,6 | 76,9 |  |
| Wide local excision | 60 | 17 | 96,5 | 76,7 |  |
| Compartment oriented excision | 25 | 6 | 91,7 | 76,7 |  |
| Amputation | 6 | 3 | 50,0 | 50,0 |  |
|  |  |  |  |  |  |
| **ILP** |  |  |  |  | 0,486 |
| no | 121 | 37 | 91,1 | 73,9 |  |
| yes | 8 | 2 | 87,5 | 87,5 |  |
|  |  |  |  |  |  |
| **Radiation** |  |  |  |  | 0,872 |
| no | 46 | 15 | 90,3 | 79,2 |  |
| yes | 83 | 24 | 91,1 | 72,6 |  |
|  |  |  |  |  |  |
| **Intention of radiation** |  |  |  |  | **<0,001** |
| neoadjuvant | 7 | 3 | 80,0 | 53,3 |  |
| adjuvant | 74 | 19 | 94,4 | 75,7 |  |
| definitive | 2 | 2 | 0,0 | 0,0 |  |
|  |  |  |  |  |  |
| **Radiation + hyperthermia** |  |  |  |  | 0,381 |
| no | 77 | 23 | 92,0 | 73,1 |  |
| yes | 6 | 1 | 83,3 | 83,3 |  |
|  |  |  |  |  |  |
| **Chemotherapy** |  |  |  |  | 0,806 |
| no | 99 | 32 | 91,8 | 75,8 |  |
| yes | 30 | 7 | 86,7 | 70,7 |  |
|  |  |  |  |  |  |
| **Intention of chemotherapy** |  |  |  |  | 0,487 |
| neoadjuvant | 20 | 5 | 86,6 | 67,3 |  |
| adjuvant | 10 | 2 | 87,5 | 75,0 |  |
|  |  |  |  |  |  |
| **Chemotherapy + hyperthermia** |  |  |  |  | 0,663 |
| no | 15 | 4 | 91,7 | 73,3 |  |
| yes | 15 | 3 | 80,8 | 69,2 |  |

Supplementary Table 1. Results of the univariate analysis regarding the overall survival (OS). (LR: local recurrence, DM: distant metastasis, ILP: isolated limb perfusion)
