# Supplementary material for: Localized Myxofibrosarcoma: A Retrospective Analysis of Primary Therapy and Prognostic Factors in 134 Patients in a Single Institution
Source: Oncologist. 2023 Dec 23;29(4):e544–52. doi: 10.1093/oncolo/oyad332 (PMC10994258; doi:10.1093/oncolo/oyad332)
Supplement: oyad332_suppl_Supplementary_Tables_2 [file oyad332_suppl_supplementary_tables_2.docx]

| **Factor (LRFS)** | **Total** | **Events (LR)** | **2-years % LRFS** | **5-years %LRFS** | **p-value** | **Factor (DMFS)** | **Total** | **Events (DM)** | **2-years % DMFS** | **5-years % DMFS** | **p-value** |
| --- | --- | --- | --- | --- | --- | --- | --- | --- | --- | --- | --- |
| **Sex** |  |  |  |  | 0,169 | **Sex** |  |  |  |  | 0,860 |
| Male | 73 | 23 | 79,5 | 73,9 |  | Male | 73 | 13 | 88,5 | 80,6 |  |
| Female | 61 | 25 | 70,0 | 56,3 |  | Female | 61 | 10 | 91,1 | 80,8 |  |
|  |  |  |  |  |  |  |  |  |  |  |  |
| **Age groups** |  |  |  |  | 0,651 | **Age groups** |  |  |  |  | 0,192 |
| ≤59 | 47 | 16 | 79,9 | 68,3 |  | ≤59 | 47 | 6 | 93,2 | 85,8 |  |
| 60 – 79 | 67 | 25 | 73,5 | 65,6 |  | 60 – 79 | 67 | 11 | 91,8 | 81,2 |  |
| ≥80 | 20 | 7 | 69,6 | 62,7 |  | ≥80 | 20 | 6 | 74,7 | 67,9 |  |
|  |  |  |  |  |  |  |  |  |  |  |  |
| **Tumor size (cT)** |  |  |  |  | 0,283 | **Local recurrence** |  |  |  |  | 0,323 |
| 1 | 34 | 7 | 84,7 | 81,2 |  | no | 86 | 12 | 92,5 | 84,0 |  |
| 2 | 47 | 12 | 81,5 | 72,6 |  | yes | 48 | 11 | 85,1 | 76,0 |  |
| 3 | 22 | 7 | 76,4 | 63,6 |  |  |  |  |  |  |  |
| 4 | 12 | 5 | 60,0 | 40,0 |  | **Tumor size (cT)** |  |  |  |  | **<0,001** |
|  |  |  |  |  |  | 1 | 34 | 2 | 93,8 | 93,8 |  |
| **Tumor size (cT)** |  |  |  |  | 0,120 | 2 | 47 | 6 | 93,2 | 85,5 |  |
| ≤2 | 81 | 19 | 82,9 | 76,3 |  | 3 | 22 | 7 | 80,2 | 63,0 |  |
| ≥3 | 34 | 12 | 71,0 | 56,5 |  | 4 | 12 | 6 | 60,6 | 36,4 |  |
|  |  |  |  |  |  |  |  |  |  |  |  |
| **Grade** |  |  |  |  | 0,798 | **Tumor size (cT)** |  |  |  |  | **<0,001** |
| 1 | 12 | 3 | 83,3 | 83,3 |  | ≤2 | 81 | 8 | 93,5 | 89,1 |  |
| 2 | 69 | 25 | 78,1 | 67,1 |  | ≥3 | 34 | 13 | 73,9 | 54,2 |  |
| 3 | 52 | 19 | 70,9 | 62,0 |  |  |  |  |  |  |  |
|  |  |  |  |  |  |  |  |  |  |  |  |
| **Grade** |  |  |  |  | 0,511 | **Grade** |  |  |  |  | 0,088 |
| 1 | 12 | 3 | 83,3 | 83,3 |  | 1 | 12 | 0 | 100,0 | 100,0 |  |
| ≥2 | 121 | 44 | 75,0 | 65,0 |  | 2 | 69 | 11 | 90,6 | 82,1 |  |
|  |  |  |  |  |  | 3 | 52 | 12 | 85,7 | 74,4 |  |
| **Grade** |  |  |  |  | 0,770 |  |  |  |  |  |  |
| ≤2 | 81 | 28 | 79,0 | 62,0 |  | **Grade** |  |  |  |  | 0,132 |
| 3 | 52 | 19 | 70,9 | 62,0 |  | ≤2 | 81 | 9 | 92,1 | 84,7 |  |
|  |  |  |  |  |  | 3 | 52 | 12 | 85,7 | 74,4 |  |
| **Localization** |  |  |  |  | 0,757 |  |  |  |  |  |  |
| Lower Extremity | 74 | 24 | 78,5 | 67,4 |  | **Localization** |  |  |  |  | 0,318 |
| Upper Extremity | 32 | 12 | 69,2 | 69,2 |  | Lower Extremity | 74 | 12 | 87,2 | 79,6 |  |
| Pelvic Region | 11 | 5 | 71,6 | 59,7 |  | Upper Extremity | 32 | 2 | 96,4 | 92,6 |  |
| Thorax | 12 | 4 | 74,1 | 63,5 |  | Pelvic Region | 11 | 2 | 90,9 | 77,9 |  |
| Other | 5 | 3 | 80,0 | 60,0 |  | Thorax | 12 | 4 | 91,7 | 61,1 |  |
|  |  |  |  |  |  | Other | 5 | 1 | 80,0 | 80,0 |  |
| **Localization** |  |  |  |  | 0,330 |  |  |  |  |  |  |
| Extremity | 106 | 36 | 75,7 | 67,8 |  | **Localization** |  |  |  |  | 0,229 |
| Other | 28 | 12 | 74,2 | 59,7 |  | Extremity | 106 | 16 | 89,9 | 83,4 |  |
|  |  |  |  |  |  | Other | 28 | 7 | 89,0 | 70,6 |  |
| **Biopsy prior to surgery** |  |  |  |  | **0,032** |  |  |  |  |  |  |
| no | 39 | 20 | 61,7 | 50,9 |  | **Biopsy prior to surgery** |  |  |  |  | 0,166 |
| yes | 95 | 28 | 81,0 | 72,7 |  | no | 39 | 4 | 92,0 | 89,1 |  |
|  |  |  |  |  |  | yes | 95 | 19 | 88,7 | 77,1 |  |
| **Clinic of biopsy** |  |  |  |  | 0,561 |  |  |  |  |  |  |
| Non-Specialists | 57 | 20 | 78,6 | 72,4 |  | **Clinic of biopsy** |  |  |  |  | 0,221 |
| Sarcoma Center | 38 | 8 | 84,3 | 72,2 |  | Non-Specialists | 57 | 10 | 89,2 | 81,5 |  |
|  |  |  |  |  |  | Sarcoma Center | 38 | 9 | 87,5 | 68,5 |  |
| **Type of biopsy** |  |  |  |  | 0,071 |  |  |  |  |  |  |
| Incision | 54 | 11 | 83,7 | 76,1 |  | **Type of biopsy** |  |  |  |  | 0,275 |
| Excision | 35 | 16 | 73,7 | 63,6 |  | Incision | 54 | 13 | 87,8 | 71,2 |  |
| Core | 6 | 1 | 100,0 | 100,0 |  | Excision | 35 | 6 | 88,0 | 81,6 |  |
|  |  |  |  |  |  | Core | 6 | 0 | 100,0 | 100,0 |  |
| **Clinic of surgery** |  |  |  |  | **0,001** |  |  |  |  |  |  |
| Non-Specialists | 35 | 21 | 61,3 | 49,9 |  | **Clinic of surgery** |  |  |  |  | **0,034** |
| Sarcoma center | 96 | 26 | 80,9 | 72,7 |  | Non-Specialists | 35 | 2 | 94,2 | 94,2 |  |
|  |  |  |  |  |  | Sarcoma center | 96 | 20 | 88,7 | 75,9 |  |
| **Resection margins** |  |  |  |  | **0,002** |  |  |  |  |  |  |
| R0 | 90 | 23 | 79,3 | 74,8 |  | **Resection margins** |  |  |  |  | 0,468 |
| R≥1 | 35 | 19 | 70,9 | 48,0 |  | R0 | 90 | 14 | 90,5 | 82,6 |  |
|  |  |  |  |  |  | R≥1 | 35 | 8 | 88,3 | 75,1 |  |
| **Type of surgery** |  |  |  |  | **<0,001** |  |  |  |  |  |  |
| LNWE | 36 | 24 | 54,0 | 42,0 |  | **Type of surgery** |  |  |  |  | 0,161 |
| WLE | 60 | 18 | 80,9 | 71,9 |  | LNWE | 36 | 4 | 91,2 | 87,9 |  |
| COE | 28 | 4 | 92,3 | 83,9 |  | WLE | 60 | 10 | 91,5 | 82,0 |  |
| Amputation | 6 | 1 | 75,0 | 75,0 |  | COE | 28 | 6 | 88,5 | 76,9 |  |
|  |  |  |  |  |  | Amputation | 6 | 2 | 75,0 | 37,5 |  |
| **ILP** |  |  |  |  | 0,111 |  |  |  |  |  |  |
| no | 126 | 47 | 74,4 | 64,6 |  | **ILP** |  |  |  |  | 0,663 |
| yes | 8 | 1 | 87,5 | 87,5 |  | no | 126 | 22 | 89,8 | 80,3 |  |
|  |  |  |  |  |  | yes | 8 | 1 | 87,5 | 87,5 |  |
| **Radiation** |  |  |  |  | **<0,001** |  |  |  |  |  |  |
| no | 49 | 26 | 52,7 | 52,7 |  | **Radiation** |  |  |  |  | 0,992 |
| yes | 85 | 22 | 87,5 | 73,6 |  | no | 49 | 8 | 86,7 | 81,6 |  |
|  |  |  |  |  |  | yes | 85 | 15 | 91,3 | 80,4 |  |
| **Intention of radiation** |  |  |  |  | **0,049** |  |  |  |  |  |  |
| neoadjuvant | 7 | 2 | 60,0 | 60,0 |  | **Intention of radiation** |  |  |  |  | **0,032** |
| adjuvant | 76 | 19 | 90,4 | 75,3 |  | neoadjuvant | 7 | 2 | 85,7 | 64,3 |  |
| definitive | 2 | 1 | 50,0 | 50,0 |  | adjuvant | 76 | 12 | 93,1 | 82,7 |  |
|  |  |  |  |  |  | definite | 2 | 1 | 50,0 | 50,0 |  |
| **Radiation + hyperthermia** |  |  |  |  | 0,534 |  |  |  |  |  |  |
| no | 79 | 21 | 88,2 | 74,0 |  | **Radiation + hyperthermia** |  |  |  |  | 0,446 |
| yes | 6 | 1 | 80,0 | 80,0 |  | no | 79 | 15 | 90,9 | 79,7 |  |
|  |  |  |  |  |  | yes | 6 | 0 | 100,0 | 100,0 |  |
| **Chemotherapy** |  |  |  |  | 0,765 |  |  |  |  |  |  |
| no | 104 | 40 | 75,5 | 66,6 |  | **Chemotherapy** |  |  |  |  | 0,968 |
| yes | 30 | 8 | 74,1 | 63,5 |  | no | 104 | 19 | 90,3 | 80,8 |  |
|  |  |  |  |  |  | yes | 30 | 4 | 86,2 | 80,5 |  |
| **Intention of chemotherapy** |  |  |  |  | 0.328 |  |  |  |  |  |  |
| neoadjuvant | 20 | 4 | 79,5 | 70,7 |  | **Intention of chemotherapy** |  |  |  |  | 0,528 |
| adjuvant | 10 | 4 | 64,3 | 51,4 |  | neoadjuvant | 20 | 2 | 91,7 | 81,5 |  |
|  |  |  |  |  |  | adjuvant | 10 | 2 | 75,0 | 75,0 |  |
| **Chemotherapy + hyperthermia** |  |  |  |  | 0,590 |  |  |  |  |  |  |
| no | 15 | 4 | 70,1 | 70,1 |  | **Chemotherapy + hyperthermia** |  |  |  |  | 0,664 |
| yes | 15 | 4 | 82,5 | 55,0 |  | no | 15 | 2 | 91,7 | 81,5 |  |
|  |  |  |  |  |  | yes | 15 | 2 | 80,0 | 80,0 |  |

Supplementary Table 2. Univariate survival analysis regarding LRFS and DMFS. (LRFS: local recurrence free survival, DMFS: distant metastasis free survival, LNWE: local non-wide excision, WLE: wide local excision, COE: compartment-oriented excision, ILP: isolated limb perfusion).
