# Supplementary material for: Localized Myxofibrosarcoma: A Retrospective Analysis of Primary Therapy and Prognostic Factors in 134 Patients in a Single Institution
Source: Oncologist. 2023 Dec 23;29(4):e544–52. doi: 10.1093/oncolo/oyad332 (PMC10994258; doi:10.1093/oncolo/oyad332)
Supplement: oyad332_suppl_Supplementary_Tables_3 [file oyad332_suppl_supplementary_tables_3.docx]

| **Site of metastasis** | **n** | **%** |
| --- | --- | --- |
| Pulmonary | 11 | 47,8 |
| Lymph nodes | 4 | 17,4 |
| Peritoneum | 2 | 8,7 |
| Soft tissue | 2 | 8,7 |
| Skeletal | 1 | 4,3 |
| Subcutis | 2 | 8,7 |
| Pleural | 1 | 4,3 |
| Total | 23 |  |

Supplementary Table 3. Sites of primary metastasis.
